# P244: Rates, microbiology and risk factors of central line associated bloodstream infection in a neonatal intensive care unit from 2003 to 2012

**DOI:** 10.1186/2047-2994-2-S1-P244

**Published:** 2013-06-20

**Authors:** C Lapresta Moros, MJ Hernández Navarrete, T Galicia Flores, JM Celorrio Pascual, VM Solano Bernad

**Affiliations:** 1 Preventive Medicine, Miguel Servet Teaching Hospital, Zaragoza, Spain

## Introduction

Central line associated bloodstream infection (CLABSI) is the most frequent healthcare-associated infection reported in neonatal intensive care units (NICU). Understanding the microbiology and risk factors of CLABSI in neonates is a key step in development of targeted prevention strategies.

## Objectives

To identify avoidable risk factors for CLABSI in neonates.

## Methods

An analytical-observational study was carried out in the NICU located in Miguel Servet Teaching Hospital, based on the data obtained from the Healthcare-associated infection Surveillance System. All neonates admitted to the unit for more than 24 hours, between April 2003 and December 2012, were eligible. The diagnosis of CLABSI was based on CDC/NHSN definitions. Infection rates were calculated and stratified according to birth weight (BW) groups (NHSN categorization). For the statistical analysis, bivariate and multivariate Cox regression models were performed.

## Results

During the study period, 1697 neonates were enrolled. 219 neonates (12.91%) had at least one CLABSI. CLABSI rates per 1000 central line-days by BW were: ≤750 g 12.54; 750-1000 g 10.808; 1001-1500 g 9.17; 1501-2500 g 4.76; >2500 g 6.74.51 and total 8.75. The most frequent isolated microorganism was coagulase-negative staphylococci (29.32%). In the bivariate and multivariate regression models, Apgar score, gestational age, duration of catheterization and parenteral nutrition showed the strongest association with CLABSI. Other factors as birth weight or rectal colonization with gram-negative bacilli were not independently associated with CLABSI.

## Conclusion

Incidence rates of CLABSI in our centre are slightly superior to the rates described in literature. Newborn baseline clinical features of the neonates enrolled for the study, defined by Apgar score and gestational age, and the use of intravascular devices are the two major determinants for endemic CLABSI.

## Disclosure of interest

None declared

